# Rapid Immunoassay for the Detection of Genital Herpes Infection

**DOI:** 10.1155/S1064744994000360

**Published:** 1994

**Authors:** Samuel M. Alexander, Bryan M. Gebhardt

**Affiliations:** 1 Women's Pavilion, Southern Baptist Hospital Louisiana State University School of Medicine 4429 Clara Street, Suite 540 New Orleans LA 70115 USA; 2 Department of Ophthalmology Louisiana State University School of Medicine New Orleans LA USA; 3 Department of Obstetrics and Gynecology Louisiana State University School of Medicine New Orleans LA USA

## Abstract

*Objective:* We evaluated a new affinity membrane strip test for the diagnosis of herpetic genital infections. Test strip results, which are available by immunoassay in 30 min without the need for special equipment, were compared with the results of viral culture.

*Methods:* Twenty-eight female patients with vulvar lesions thought to be due to genital herpes simplex virus (HSV) infection were tested. The affinity membrane strip was applied to the genital lesion. Dacron swabs were then applied to the lesions and the swab contents cultured for HSV. For the immunoassay, the test strip was immersed in peroxidase-labeled antibodies specific for HSV type 2 (HSV-2), incubated, washed, and developed in the substrate tetramethylbenzidine. Positive reactions appeared as intense blue spots roughly the shape and size of the lesion. Sensitivity and specificity of the test were determined using the results of viral cultures as the standard.

*Results:*The lesions of 8 patients yielded positive strips that correlated with positive cultures. The lesions of 6 patients produced positive strips but the cultures were negative. None of the patients whose lesions yielded negative strips had positive cultures. The lesions of 14 patients produced negative strips and negative cultures. Strips and viral cultures from 10 control patients (no lesions) were negative. Sensitivity and specificity were 100% and 70%, respectively. Positive (PPV) and negative (NPV) predictive values were 57% and 100%, respectively. The accuracy was 78%.

*Conclusions:* The affinity membrane test was extremely sensitive in detecting herpetic genital infection compared with viral culture. The specificity was lower, resulting from false positives based on negative cultures. However, negative cultures may occur in the presence of disease, depending in part on the type of lesion.  Thus, specificity may be higher than this preliminary study indicates, and more elaborate search for virus including serologic studies as well as larger groups of patients may be needed to refine this evaluation. With further testing and development,  this membrane affinity test for herpes may yield a valuable adjunct to clinical diagnosis of this infection.


Infectious Diseases in Obstetrics and Gynecology 2:30–33 (1994)
© 1994 Wiley-Liss, Inc.

### Rapid Immunoassay for the Detection of Genital Herpes Infection

**Samuel M. Alexander and Bryan M. Gebhardt**

*Women's Pavilion, Southern Baptist Hospital (S.M.A.), and Departments of Ophthalmology (B.M.G.) and Obstetrics and Gynecology (S.M.A.), Louisiana State University School of Medicine, New Orleans, LA*

### Abstract

**Objective:** We evaluated a new affinity membrane strip test for the diagnosis of herpetic genital infections. Test strip results, which are available by immunoassay in 30 min without the need for special equipment, were compared with the results of viral culture.

**Methods:** Twenty-eight female patients with vulvar lesions thought to be due to genital herpes simplex virus (HSV) infection were tested. The affinity membrane strip was applied to the genital lesion. Dacron swabs were then applied to the lesions and the swab contents cultured for HSV. For the immunoassay, the test strip was immersed in peroxidase-labeled antibodies specific for HSV type 2 (HSV-2), incubated, washed, and developed in the substrate tetramethylbenzidine. Positive reactions appeared as intense blue spots roughly the shape and size of the lesion. Sensitivity and specificity of the test were determined using the results of viral cultures as the standard.

**Results:** The lesions of 8 patients yielded positive strips that correlated with positive cultures. The lesions of 6 patients produced positive strips but the cultures were negative. None of the patients whose lesions yielded negative strips had positive cultures. The lesions of 14 patients produced negative strips and negative cultures. Strips and viral cultures from 10 control patients (no lesions) were negative. Sensitivity and specificity were 100% and 70%, respectively. Positive (PPV) and negative (NPV) predictive values were 57% and 100%, respectively. The accuracy was 78%.

**Conclusions:** The affinity membrane test was extremely sensitive in detecting herpetic genital infection compared with viral culture. The specificity was lower, resulting from false positives based on negative cultures. However, negative cultures may occur in the presence of disease, depending in part on the type of lesion. Thus, specificity may be higher than this preliminary study indicates, and more elaborate search for virus including serologic studies as well as larger groups of patients may be needed to refine this evaluation. With further testing and development, this membrane affinity test for herpes may yield a valuable adjunct to clinical diagnosis of this infection. © 1994 Wiley-Liss, Inc.

Key words

Antibody, diagnosis, membrane affinity strip, virus

Genital herpes is a common sexually transmitted disease that occurs in all social classes and in all age groups. More than 85% of all genital herpes infections are caused by herpes simplex virus type 2 (HSV-2), which differs only slightly from type 1 (HSV-1), a common cause of cold sores of the mouth.[1]

Genital herpes is characterized by vesicles on the mucous membranes as well as on the external genitalia. On most surfaces, the vesicles are present transiently and rapidly convert to superficial ulcerations rimmed by an inflammatory infiltrate. The diagnosis is often based on the presence of painful shallow vulvar ulcers. Viral cultures obtained from

Address correspondence/reprint requests to Dr. Samuel M. Alexander, Women's Pavilion, Southern Baptist Hospital, 4429 Clara Street, Suite 540, New Orleans, LA 70115.

Received January 27, 1994
Accepted May 16, 1994

Clinical Study these lesions aid in confirming the diagnosis. However, the results of culture are not usually available for several days to weeks.[1,2] Microscopic evaluation of Papanicolaou smears from the lesions reveals multinucleate giant cells, as well as cells with large intranuclear inclusions that are characteristic features of the herpes virus infection.

The value of a rapid and accurate diagnosis of HSV-2 infection cannot be overemphasized. The stigma and implications of this diagnosis are very frightening. During pregnancy, there is an increased risk of infecting the fetus if a vaginal delivery should occur in a patient with active herpes infection. Thus, there is need for a rapid and accurate diagnosis of this infection in near-term pregnancies.[2,3]

We designed a new test to diagnose herpetic genital lesions. We report here a preliminary study of the usefulness of this method in comparison with viral culture.

### SUBJECTS AND METHODS

### Patients

Twenty-eight females with vulvar lesions suspicious for genital herpes were enrolled in the study. These patients were divided into those with primary lesions (N = 13) and those with recurrent lesions (N = 15). The lesions ranged in size from 2 to 12 mm across. The duration of lesions at the time of the clinic visit ranged from 1 to 6 days, and no other infections were evident at this time. Ten normal female patients (no lesions) were tested as controls.

### Affinity Membrane Test/Viral Culture

The affinity membrane test makes use of a synthetic protein-binding membrane (Immunodyne Immunoaffinity Membrane, Pall Corporation, East Hills, NY). Strips of affinity membrane (1.5 × 8 cm) were sterilized by ethylene oxide. For use, an affinity membrane strip was applied to the vulvar lesion and then placed in a snap-cap plastic test tube or Ziploc bag until the immunoassay was completed. In the case of the normal control patients, the affinity membrane strips were applied to the normal vulvar epithelium before processing.

The order of test strip application and specimen collection for culture was alternated in successive patients; sampling with the membrane affininty strip was followed by sampling for viral culture in one patient and the procedures reversed in the next patient.

Affinity membrane strips were processed according to the following protocol. Individual strips were immersed in a solution of 0.1% sodium azide and 0.3% hydrogen peroxide for 5 min to inactivate endogenous peroxidase activity due to inflammatory cells present in the lesions. The strip was then rinsed once in deionized water and immersed in 10 ml of 0.5% casein (Diffco Laboratories, Detroit, MI) in phosphate-buffered saline (PBS) to which was added a 1:1,000 dilution of rabbit anti-HSV-2 antibody conjugated with horseradish peroxidase (Dako Corporation, Carpenteria, CA). The strip was incubated in the casein/antibody solution for 5 min at room temperature. Next, the strip was washed for three 2-minute intervals in PBS containing 1% Triton-X detergent, then washed twice in deionized water and immersed in the tetramethylbenzidine/hydrogen peroxide substrate solution (Kirkergaard & Perry Laboratories, Inc., Gaithersburg, MD). In positive tests, color development began rapidly and was essentially complete within 5 min; a dark-blue staining pattern reflecting the size and shape of the lesion developed where the viral antigens on the test strip had bound the anti-herpes antibody. On completion of color development, the test strip was removed from the substrate solution, rinsed in deionized water, dried, and retained as a permanent record of results in the patient's chart.

To obtain culture specimens, we applied sterile Dacron swabs to the lesion or to normal vulvar epithelium (controls). Each swab was individually placed in a sterile capped test tube containing 2 ml of viral transport medium and stored for no longer than 8 h at 4°C until processing. For viral culture, the swab was withdrawn from the viral transport medium and pressed against the side of the tube. Then the swab and 0.1-ml quantities of viral transport medium were transferred to separate tissue culture tubes containing monolayers of CV-1 viral indicator cells and incubated at 37°C in a $CO_2$ incubator. The swab was removed from the viral culture tube 24 h later. Viral culture tubes were observed every 2 days for a total of 21 days. The presence of cytopathic effect (CPE) was recorded using an inverted tissue culture microscope. The technician scoring the CPE in the cell cultures was

**TABLE 1. Results of affinity membrane strip test compared with viral culture[a]**

| | Disease (culture results) | |
|---|---|---|
| | Positive | Negative |
| Test strip results | | |
| Positive | 8 (true positives) | 6 (false positives) |
| Negative | 0 (false negatives) | 14 (true negatives) |

[a]Sensitivity = 100%, specificity = 70%, PPV = 57%, NPV = 100%, and accuracy = 78%.

masked as to the status of the patients to eliminate possible bias.

### RESULTS

Clinically, the lesions observed were thought to be consistent with the diagnosis of genital herpes infection. Of the 28 patients, 8 yielded positive strips that correlated with positive viral cultures and 6 had positive strips and negative viral cultures. None of the patients whose strips were negative had positive cultures. The lesions of 14 patients produced negative strips and negative viral cultures. Based on these numbers, sensitivity was 100%, specificity was 70%, positive (PPV) and negative (NPV) predictive values were 57% and 100%, respectively, and accuracy was 78% (Table 1). All 10 control strips and cultures were negative.

### DISCUSSION

Accurate diagnosis of genital herpes is essential, especially when the social and perinatal implications of this infection are considered. Diagnosis of genital herpes has been made by physical findings and patient history. The most reliable method for HSV diagnosis has been viral culture.[2] Tissue cultures are generally observed for 7–10 days to determine if cytopathic changes have occurred. However, the success of viral isolation from the lesion may depend upon the stage of the herpetic lesion. Various studies have shown that HSV could be isolated from only 27% of crusted lesions, 70% of ulcers, 87% of pustular lesions, and 94% of vesicles.[2–4] Thus, there might be some difficulty in isolating HSV from a very suspicious lesion, and a negative culture does not necessarily rule out the presence of genital herpes. In our study, the inability to isolate HSV in tissue culture in 6 patients who had positive strips might have been due to the state of the lesion. The fact that there were no patients who had a positive culture with a negative strip supports this possibility. We did not collect data on the type of lesion, so no correlation is possible here, but larger studies planned for the future will attempt to incorporate this information.

Cytologic evaluation has been utilized in the diagnosis of genital herpes. Tzank preparations of the scraped lesions demonstrated the presence of multinucleate giant cells. However, this method can produce false negative and false positive results. Landy and Grossman[2] stated in their review that positive exfoliative cytology was found in only 38–50% of HSV culture-positive patients. Thus, there is a need for a more sensitive and specific rapid test.

Serologic testing to detect the presence of HSV antibodies has been used for diagnosis. Differentiation between antibodies for HSV-1 and HSV-2 is still quite difficult. Koutsky et al.[5] stated that HSV-2 serologic testing is more useful than viral culture in confirming the diagnosis of genital HSV-2 infection among women with recurrent HSV-2 infection, based on isolation of HSV-2 antibodies in 37 of 38 patients by serology compared with successful viral isolation in only 34%. This study also reaffirms the difficulties inherent in isolating HSV-2 in viral culture.

Background investigation and testing of our new membrane affinity test were originally conducted on animal models. Gebhardt et al.[6,7] utilized the affinity membrane strip for the detection of herpetic keratitis with great specificity. The membrane affinity test is not only a simple and rapid test, but it does not require specialized equipment or training to complete. The results of the current preliminary study, although involving only a small number of patients, appear to demonstrate that the rapid affinity membrane test could be a valuable tool in the diagnosis of genital herpes infections. The difficulty in isolating HSV-2 from viral culture suggests that perhaps another method should be used to diagnose HSV-2 lesions. Further studies are planned to compare the results of this test with those of serologic studies as well as viral culture.

### ACKNOWLEDGMENTS

This work was supported in part by U.S. Public Health Service grants EY08701, EY02672, and

EY02377 from the National Eye Institute, National Institutes of Health, Bethesda, MD.

### REFERENCES

1. Sweet RL, Gibbs RS: Infectious Diseases of the Genital Tract. Vol 2. Baltimore: Willams & Wilkins, pp 183–188, 1985.
2. Landy HJ, Grossman JH: Herpes simplex virus. Obstet Gynecol Clin North Am 16:495–511, 1989.
3. Kulhanjian JA, Soroush V, Au DS, et al.: Identification of women at unsuspected risk of primary infection with herpes simplex virus type 2 during pregnancy. N Engl J Med 326:916–920, 1992.
4. Corey L, Adams HG, Brown ZA, Holmes KK: Genital HSV infection: Clinical manifestations, course, and complications. Ann Intern Med 98:958–972, 1983.
5. Koutsky LA, Stevens CE, Holmes KK, et al.: Underdiagnosis of genital herpes by current clinical and viral-isolation procedures. N Engl J Med 326:1533–1539, 1992.
6. Gebhardt BM, Rootman DS, Reidy JJ, Kaufman HE: Rapid confirmation of corneal herpetic infection with an affinity membrane test. American Academy of Ophthalmology Abstract. Ophthalmology 95 (Suppl):159, 1988.
7. Gebhardt BM, Reidy JJ, Kaufman HE: An affinity membrane test for superficial corneal herpes. Am J Ophthalmol 105:686–687, 1988.
